# Peripheral blood absolute lymphocyte/monocyte ratio recovery during ABVD treatment cycles predicts clinical outcomes in classical Hodgkin lymphoma

**DOI:** 10.1038/bcj.2013.8

**Published:** 2013-04-19

**Authors:** L F Porrata, K M Ristow, T M Habermann, W R Macon, T E Witzig, J P Colgan, D J Inwards, S M Ansell, I N Micallef, P B Johnston, G Nowakowski, C A Thompson, S N Markovic

**Affiliations:** 1 Division of Hematology, Department of Internal Medicine, Mayo Clinic College of Medicine, Mayo Clinic, Rochester, MN, USA; 2 Division of Hematopathology, Department of laboratory Medicine and Pathology, Mayo Clinic, Rochester, MN, USA

**Keywords:** classical Hodgkin lymphoma, survival ALC/AMC ratio, ABVD chemotherapy

## Abstract

The peripheral blood absolute lymphocyte/monocyte count ratio at diagnosis (ALC/AMC-DX) predicts survival in classical Hodgkin lymphoma (cHL). However, a limitation of the ALC/AMC-DX is the inability to assess sequentially the host/tumor interaction during treatment. Therefore, we *retrospectively examined* the ALC/AMC ratio, as a surrogate marker of host immunity (ALC) and tumor microenvironment (AMC), at each adriamycin, bleomycin, vinblastine and dacarbazine treatment cycle as a predictor for clinical outcomes. From 1990 until 2008, 190 cHL patients were diagnosed, treated and followed at Mayo Clinic Rochester and qualified for the study. The ALC/AMC ratio at each treatment cycle was a predictor for overall survival (OS) and progression-free survival (PFS). An ALC/AMC ratio ⩾1.1 versus ALC/AMC <1.1 during treatment cycles was an independent predictor for OS (hazard ratio (HR)=0.14; 95% confidence interval (CI): 0.04–0.40; *P*<0.0002) and for PFS (HR=0.19; 95% CI: 0.05–0.82; *P*<0.03). The ALC/AMC ratio during treatment cycles is a predictor for survival and provides a platform to develop therapeutic modalities to manipulate the ALC/AMC ratio during chemotherapy to improve clinical outcomes in cHL.

## Introduction

The peripheral blood absolute lymphocyte/monocyte count ratio at diagnosis (ALC/AMC-DX) has been recently reported and confirmed to be a predictor for clinical outcomes in classical Hodgkin lymphoma (cHL).^1, 2^ An inverse correlation has been reported between the ALC/AMC-DX and tumor-associated macrophages in cHL, suggesting an association between the host biological response in the macroenvironment (peripheral blood) and microenvironment (tumor bed).^2^ However, a limitation of the ALC/AMC-DX, the International Prognostic Score at diagnosis and the interim positron emission tomography (int-PET-scan) is the inability to assess sequentially the host/tumor interaction during treatment, as they are performed at one specific time point during the initial treatment course of cHL patients. Therefore, we studied the ALC/AMC ratio recovery, as a surrogate marker of host immunity (that is, ALC) and tumor microenvironment (that is, AMC), at each treatment cycle phase in patients treated with adriamycin, bleomycin, vinblastine and dacarbazine (ABVD) to assess its predictive ability for clinical outcomes in cHL.

## Materials and methods

### Patients

To participate in this study, patients were required to have newly diagnosed cHL, treated with ABVD with or without radiation and be followed at the Mayo Clinic (Rochester, MN, USA). Patients diagnosed with nodular lymphocyte -predominant HL (NLPHL), treated only with radiation or palliative care, positive for human immunodeficiency virus or with concomitant autoimmune disease receiving immunosuppressive therapy were excluded. From 1990 to 2008, 190 consecutive cHL qualified for the study. No patients refused authorization to use their medical records for research and none were lost to follow-up. Approval for the retrospective review of these patients' records was obtained from the Mayo Clinic Institutional Review Board, and the research was conducted in accordance with the USA Federal Regulations and the Declaration of Helsinki.

### End point

The primary end point of the study was to assess the impact of the ALC/AMC ratio recovery during each cycle phase of ABVD therapy on overall survival (OS) and progression-free survival (PFS) from cHL diagnosis.

The cutoff for the ALC/AMC ratio ⩾1.1 used in this study was based on our previous publication^1^ and obtained from the complete blood cell count^3^ from a similar automated technology with devices that have included the Coulter STKR, Coulter STKS, Coulter GENS, Coulter LH500, Coulter LH750, Coulter HmX, Coulter AcT Diff5, Sysmex XE5000, Sysmex XE2100, Sysmex1800 and Sysmex 2000 at each cycle phase of ABVD treatment. The ALC/AMC ratio was obtained by dividing the ALC over the AMC from the complete blood cell count at each cycle phase of ABVD chemotherapy.

### Prognostic factors

The prognostic factors evaluated in the study included the International Prognostic Score (IPS)^4^ at diagnosis for advanced stage patients: age >45 years, albumin <4 g/dl, ALC <600/μl or <8% of white cell count, hemoglobin <10.5 g/dl, male gender, stage IV and white blood cell count ⩾15 000/μl; limited versus advanced stage; treatment modality (combination chemotherapy plus radiation versus chemotherapy alone), int-PET-scan and the ALC/AMC ratio recovery during each cycle phase of ABVD chemotherapy.

### Response and survival

Definitions of response criteria, OS and PFS were based on the guidelines from the International Harmonization Project Lymphoma.^5^ OS was defined as the time from cHL diagnosis to death as a result of any cause or last follow-up. PFS was defined as the time from cHL diagnosis to the time of progression, relapse from complete response, death as a result of any cause or last follow-up. Patients without an event or death were censored at the time of last known follow-up.

### Statistical analysis

OS and PFS were analyzed using the approach of Kaplan and Meier.^6^ Differences between survival curves were tested for statistical significance using the two-tailed log-rank test. The Cox proportional hazard model^7^ was used for the univariate and multivariate analyses to evaluate the variables under the ‘Prognostic factors' section to assess their impact on OS and PFS. Chi-squared tests were used to determine relationships between categorical variables. The Wilcoxon's rank-sum test was used to determine associations between continuous variables and categories, and Spearman's correlation coefficients were used to evaluate associations for continuous variables. All *P*-values are two-sided associations and *P*-values <0.5 are considered statistically significant.

## Results

### Patients' characteristics

The median age at diagnosis for this cohort of 190 cHL patients was 36 years (range: 18–83 years). The distribution of additional baseline characteristics for these patients is presented in Table 1. The median follow-up for the cohort was 3.7 years (range: 0.2–20 years) and for the living patients (*N*=163) it was 4.6 years (range: 0.5–20 years). Thirteen patients died of causes not related to lymphoma or the treatment of lymphoma, and 14 patients died secondary to relapse/progression of cHL. Forty-one percent (77/190) of patients, and their ALC/AMC ratio at diagnosis was used for cycle 1A.

### ALC/AMC ratio recovery at each treatment cycle and survival

**Figure 1:**
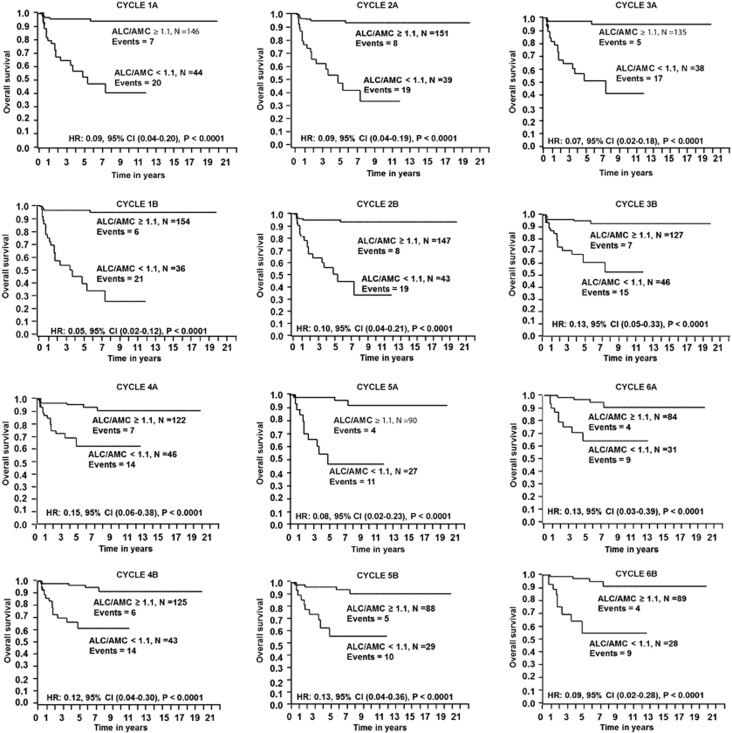
OS based on the ALC/AMC ratio at each treatment cycle phase.

Kaplan–Meier analysis was used to study OS and PFS based on ALC/AMC ratio at each treatment cycle phase of ABVD. Patients with an ALC/AMC ratio ⩾1.1 experienced superior OS compared with patients with an ALC/AMC ratio <1.1 at each treatment cycle phase of ABVD (Figure 1 and see Supplementary File). Similarly, patients with an ALC/AMC ratio ⩾1.1 at each treatment cycle experienced superior PFS (see Supplementary Figure 1).

### Number of cycles with an ALC/AMC ratio ⩾1.1 and survival

**Figure 2:**
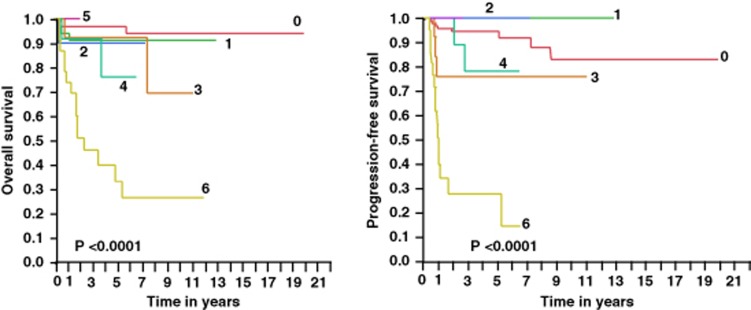
OS, lymphoma-specific survival, PFS and time to progression based on the number of treatment cycles phases that achieved an ALC/AMC ratio ⩾1.1. Worst clinical outcome observed in patients were the ALC/AMC ratio <1.1 in all treatment cycles phases. 0=all cycles with an ALC/AMC ratio ⩾1.1, *N*=96, events=4; 1=1 cycle with an ALC/AMC ratio <1.1, *N*=34, events=3; 2=2 cycles with an ALC/AMC ratio <1.1, *N*=10, events=1; 3=3 cycles with an ALC/AMC ratio <1.1, *N*=13, events=2; 4=4 cycles with an ALC/AMC ratio <1.1, *N*=12, events=2; 5=5 cycles with an ALC/AMC ratio <1.1, *N*=2, events=0; and 6=all cycles with an ALC/AMC ratio <1.1, *N*=23, events=15.

To further understand the significance of the ALC/AMC ratio ⩾1.1 recovery during each treatment cycle phase, we categorized patients according to how many cycles the ALC/AMC ratio ⩾1.1 was observed. We observed a progressive worsening of OS, lymphoma-specific survival, PFS and time to progression the more treatment cycles patients did not achieve an ALC/AMC ratio ⩾1.1 (Figure 2). Specifically, patients with an ALC/AMC ratio <1.1 during all treatment cycle phases experienced the worst OS and PFS.

### ALC/AMC ratio ⩾1.1 during any treatment cycle versus ALC/AMC ratio <1.1 during all treatment cycles

From the Kaplan–Meier curves in Figure 2, patients with an ALC/AMC ratio <1.1 in all treatment cycles separated from patients with an ALC/AMC ratio ⩾1.1 in any treatment cycle with regard to OS and PFS. Thus, patients were divided into two groups: ALC/AMC ratio ⩾1.1 in any treatment cycle versus ALC/AMC ratio <1.1 in all treatment cycles (Table 2). The differences between the groups were ALC at diagnosis, albumin, IPS risk factors and int-PET-scan. Both groups were balanced with regard to how many treatment cycles were given. In the ALC/AMC ratio ⩾1.1 in any cycle, 67% (8/12) patients experienced an unrelated lymphoma death versus 33% (5/15) in the ALC/AMC ratio <1.1 in all cycles (*P*=0.1).

**Figure 3:**
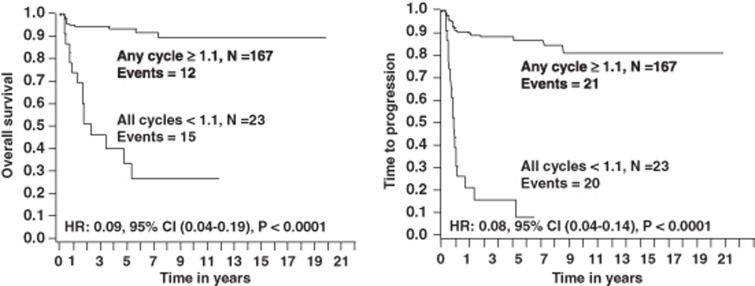
OS, lymphoma-specific survival, PFS and time to progression based on patients with an ALC/AMC ratio ⩾1.1 in any treatment cycles phases compared with patients with an ALC/AMC <1.1 in all treatment cycle phase.

Patients with an ALC/AMC ratio ⩾1.1 during any treatment cycle experienced superior OS and PFS (Figure 3) compared with patients with an ALC/AMC ratio <1.1 during all treatment cycles (OS: median was not reached versus 2.3 years, the 5-year OS rates were 93% (95% confidence interval (CI): 89–98%) versus 27% (95% CI: 10–52%) (*P*<0.0001), respectively; and PFS: median was not reached versus 0.8 years, the 5-year PFS rates were 88% (95% CI: 79–95%) versus 8% (95% CI: 5–39%) (*P*<0.0001), respectively).

### Univariate and multivariate analyses

In the univariate analysis by the Cox model, the ALC/AMC ratio ⩾1.1 at each treatment cycles as well as ALC/AMC ratio ⩾1.1 during any treatment cycle were predictors for OS and PFS (Table 3). In the multivariate analysis by the Cox model, the ALC/AMC ratio ⩾1.1 during any treatment cycle remained an independent predictor for OS and PFS (Table 4).

## Discussion

The ALC/AMC ratio, as a surrogate marker of host immunity (that is, ALC) and tumor microenvironment (that is, AMC), is a predictive biomarker for clinical outcomes in cHL. However, a limitation of the ALC/AMC-DX is its inability to assess the host/tumor interaction during treatment as it is performed at one point in time. Therefore, we analyzed the ALC/AMC ratio during treatment to assess its role on clinical outcomes in cHL.

To support the hypothesis that the biomarker ALC/AMC ratio affects survival in cHL during treatment, we evaluated the OS and PFS based on the ALC/AMC ratio ⩾1.1 during each treatment cycle phase of ABVD chemotherapy. An ALC/AMC ratio ⩾1.1 observed during each treatment cycle phase of ABVD was independently associated with superior OS and PFS. To evaluate the significance of ALC/AMC ratio recovery during each treatment cycle phase independently, we evaluated the impact on clinical outcomes based on the number of cycles that the ALC/AMC ratio was greater or not than 1.1. The more cycles with an ALC/AMC ratio <1.1, the more inferior the OS and PFS were observed, specifically in patients with an ALC/AMC ratio <1.1 in all treatment cycles. Because patients with an ALC/AMC ratio <1.1 in all treatment cycles separated dramatically with regard to clinical outcomes compared with the other patient groups, we, then, proceeded to dichotomize patients into patients with an ALC/AMC ratio ⩾1.1 in any treatment cycle versus patients with an ALC/AMC ratio <1.1 in all treatment cycles. Using this simplified grouping, patients with an ALC/AMC ratio ⩾1.1 during any treatment cycle experienced superior OS and PFS compared with patients with an ALC/AMC ratio <1.1 during all treatment cycles. By the Cox model multivariate analysis, the ALC/AMC ratio ⩾1.1 during any treatment cycle was an independent predictor for OS and PFS compared with other prognostic factors. Furthermore, patients with an ALC/AMC ratio <1.1 in all treatment cycles tended to have more adverse features including a higher number of risk factors based on the IPS and higher incidence of positive PET-scan, suggesting an impact of host immunity (that is, ALC) versus tumor microenvironment (that is, AMC) on tumor growth control.

Several studies have reported ALC recovery during the initial standard therapy in patients with acute lymphoblastic leukemia,^8^ acute myelogenous leukemia,^9^ and non-HL,^10^ suggesting that the host immune status during treatment might have a direct impact of the patient prognosis and survival. Furthermore, in diffuse large B-cell lymphoma (DLBCL) the published absolute monocyte/lymphocyte prognostic score was found to be independent prognostic factor of survival when compared with the cell of origin in DLBCL and the absolute monocyte/lymphocyte prognostic score was able to further discriminate clinical outcomes in patients with either activated B-cell or germinal center DLBCL.^11^ In cHL, gene-expression profiling studies have reported that tumor-infiltrating myeloid-derived cells predict clinical outcomes in cHL^12^ However, a practical clinical limitation of gene-expression profiling is fresh frozen tissue samples to analyze. In patients who achieve a complete response during treatment, no tumor samples are available to biopsy to provide a dynamic real-time interaction between host response and tumor. Tumor-associated macrophages are derived from circulating monocytes and recruited to the tumor site by soluble tumor-derived chemotactic factors.^13, 14, 15, 16^ The ALC/AMC-DX as a surrogate marker of host immunity (that is, ALC) and tumor microenvironment (that is, AMC) has been reported as a prognostic biomarker of clinical outcomes in cHL.^1, 2^ However, the ALC/AMC-DX is only obtained at diagnosis and it does not provide a sequential assessment of the host/tumor interaction during treatment. Therefore, the ALC/AMC ratio was analyzed during treatment to assess implications on prognosis and survival. This study demonstrated that patients maintaining a high ALC/AMC ratio during treatment experienced better clinical outcomes compared with those who did not. These observations suggest that the surrogate markers of the interaction between host immunity and tumor microenvironment not only at diagnosis but also during treatment directly impact survival in cHL using a dynamic real-time biomarker in the ALC/AMC ratio.

To minimize the inherent biases due to the nature of retrospective studies, the following steps were taken. With regards to selection bias, we only included patients with cHL and excluded any patient with NLPHL, as NLPHL is considered to be a different disease entity. Only patients treated with ABVD chemotherapy with or without subsequent radiation therapy were included as this chemotherapy regimen is currently considered the standard of care in North America. Therefore, we excluded any patient treated up-front with palliative care or radiation therapy alone, as chemotherapy with or without radiation is considered also the current standard of care in cHL. With regard to confounding factors, our study included currently used clinical prognostic factors such as the IPS and int-PET-scan. In the multivariate analysis, the ALC/AMC ratio during treatment cycles remained an independent predictor for survival when compared with these clinical prognostic factors.

The strength of the study is the follow-up of a well-defined group of patients with cHL with a median follow-up of 3.7 years for the entire cohort of patients and 4.6 years for living patients. Second, the ALC/AMC ratio combines the clinical surrogate biomarkers for the inflammatory, pathological biomarkers—tumor-infiltrating lymphocytes and tumor-associated macrophages—which directly affect the biology of cHL. In addition, an inverse correlation has also been reported between the ALC/AMC ratio and tumor-associated macrophages in cHL, suggesting an association between the host biological response in the macroenvironment (peripheral blood) and microenvironment (tumor bed).^2^ Third, the ALC/AMC ratio is a simple, easily determined clinical biomarker that can be used to assess the clinical outcomes in cHL patient at any time during the course of treatment.

In conclusion, the ALC/AMC ratio recovery during treatment cycles in cHL is prognostic biomarker for clinical outcomes and provides a platform to develop therapeutic intervention to manipulate the ALC/AMC ratio during the treatment to improve clinical outcomes in cHL. Further studies are warranted to confirm our findings.

## Figures and Tables

**Table 1:** Baseline patients' characteristics

*Characteristics*	N (*%*)	*Median*	*Range*
Age (years)	190 (100)	36	18–83
			
*Gender*			
Male	95 (50)		
Female	95 (50)		
			
WBC × 10^9^/l at diagnosis	190 (100)	8.7	1.8–53.9
ALC × 10^9^/l at diagnosis	190 (100)	1.27	0.15–9.1
Hgb (g/dl)	190 (100)	12.9	8.3–17.2
Albumin (g/dl)	162 (85)	4	1.9–5.8
			
*Stage*			
I	12 (6)		
II	80 (42)		
III	57 (57)		
IV	41 (22)		
			
*Initial treatment*			
CT+RT	84 (44)		
CT	106 (56)		
			
*IPS*			
Age (years			
>45	66 (33)		
⩽45	124 (65)		
Albumin (g/dl) (*N*=162			
⩾4	88 (54)		
<4	74 (46)		
Hgb (g/dl			
>10.5	161 (85)		
⩽10.5	29 (15)		
WBC × 10^9^/l			
>15	22 (12)		
⩽15	168 (88)		
ALC × 10^9^/l			
<0.6	19 (10)		
⩾0.6	171 (90)		
Male	95 (50)		
Stage 4	41 (22)		
IPS factors index			
⩾3	48 (25)		
<3	142 (75)		
PET-scan (*N*=111			
Positive	13 (12)		
Negative	98 (88)		
Number of cycles given			
2	17 (9)		
3	5 (3)		
4	51 (26)		
5	5 (3)		
6	112 (29)		
Cycle 1A			
ALC/AMC ratio	190 (100)	2.01	0.15–85.5
Cycle 1B			
ALC/AMC ratio	190 (100)	2.24	0.22–37.5
Cycle 2A			
ALC/AMC ratio	190 (100)	1.98	0.22–60.2
Cycle 2B			
ALC/AMC ratio	190 (100)	1.87	0.10–26.5
Cycle 3A			
ALC/AMC ratio	173 (91)	1.91	0.15–20.8
Cycle 3B			
ALC/AMC ratio	173 (91)	1.67	0.15–9.8
Cycle 4A			
ALC/AMC ratio	168 (88)	1.71	0.20–19.4
Cycle 4B			
ALC/AMC ratio	167 (88)	1.80	0.24–5.8
Cycle 5A			
ALC/AMC ratio	118 (62)	1.60	0.24–9.3
Cycle 5B			
ALC/AMC ratio	118 (62)	1.74	0.25–14.2
Cycle 6A			
ALC/AMC ratio	115 (61)	1.66	0.30–5.0
Cycle 6B			
ALC/AMC ratio	114 (60)	1.61	0.36–9.8

Abbreviations: ALC, absolute lymphocyte count; AMC, absolute monocyte count; CT, chemotherapy; Hgb, hemoglobin; IPS, International Prognostic Score; PET, positron emission tomography; RT, radiation therapy; WBC, white blood cell count.

**Table 2:** Baseline patients' characteristics based on any cycle with an ALC/AMC ratio ⩾1.1 versus all cycles with an ALC/AMC ratio <1.1

*Variables*	*Any cycle with an ALC/AMC ratio ⩾1.1*	*All cycles with an ALC/AMC ratio <1.1*	P*-value*
Age (years), median (range)	36 (18–79)	49 (18–83)	0.1
			
*Gender*			0.2
Female	87 (52.1%)	8 (34.8%)	
Male	80 (47.9%)	15 (65.2%)	
			
ALC < × 10^9^/l at diagnosis, median (range)	1.35 (0.15–3.63)	0.69 (0.27–9.1)	<0.0002
Albumin (g/dl), median (range) (*N*=162)	4.05 (1.9–5.8)	3.8 (2.3–4.1)	<0.009
Hgb (g/dl), median (range)	12.9 (8.3–17.2)	12.4 (8.8–14.3)	0.2
AMC at diagnosis × 10^9^/l	0.65 (0.14–1.63)	1.11 (0.21–2.61)	<0.0001
			
*Stage*			0.3
Limited	81 (48.5%)	8 (34.8%)	
Advanced	86 (51.5%)	15 (65.2%)	
			
WBC × 10^9^/l	8.7 (1.8–53.9)	9.7 (4.4–18.2)	0.4
*Initial treatment*			0.2
CT	108 (64.7%)	18 (78.3%)	
CT+RT	59 (35.3%)	5 (21.7%)	
			
*IPS risk factors*			
Age (years)			0.1
>45	113 (67.7%)	12 (52.2%)	
⩽45	54 (32.3%)	11 (47.8%)	
Albumin (g/dl) (*N*=162)			<0.03
⩾4	82 (57.8%)	6 (30%)	
<4	60 (42.3%)	14 (70%)	
ALC per μl			<0.0001
⩾600	157 (94%)	14 (60.9%)	
<600	10 (6%)	9 (39.1%)	
Hgb (g/dl)			0.8
>10.5	142 (85%)	19 (82.6%)	
⩽10.5	25 (15%)	4 (17.4%)	
WBC × 10^9^/l			0.5
>15	21 (12.6%)	1 (11.6%)	
⩽15	146 (87.4%)	22 (88.4%)	
Stage 4			0.6
Yes	35 (21%)	6 (26.1%)	
No	132 (79%)	17 (73.9%)	
Number of IPS risk factors			<0.03
0	21 (12.5%)	2 (8.7%)	
1	61 (36.5%)	4 (17.4%)	
2	50 (30.0%)	4 (17.4%)	
3	18 (10.8%)	8 (34.8%)	
4	13 (7.8%)	3 (13.0%)	
5	3 (1.8%)	2 (8.7%)	
6	1 (0.06%)	0 (0.0%)	
IPS factors index			<0.0006
⩾3	35 (21%)	13 (56.5%)	
<3	132 (79%)	10 (43.5%)	
Radiation			0.2
Yes	5(35.3%)	5 (21.7%)	
No	108 (64.7%)	18 (78.3%)	
PET-scan			<0.0003
Positive	7 (7%)	6 (54.6%)	
Negative	93 (93%)	5 (45.4%)	
Number of cycles given			0.6
2	15 (9%)	2 (9%)	
3	5 (3%)	0 (0%)	
4	15 (27%)	6 (26%)	
5	5 (3%)	0 (0%)	
6	97 (58%)	15 (65%)	

Abbreviations: ALC, absolute lymphocyte count; AMC, absolute monocyte count; CT, chemotherapy; Hgb, hemoglobin; IPS, International Prognostic Score; PET, positron emission tomography; RT, radiation therapy; WBC, white blood cell count.

**Table 3:** Univariate analysis for OS, lymphoma-specific survival, PFS and time to progression

*Variables*	*OS*			*PFS*		
	*HR*	*95% CI*	P*-value*	*HR*	*95% CI*	P*-value*
Age >45 years	5.72	2.58–13.90	<0.0001	2.61	1.41–4.89	<0.002
Albumin <4 g/dl	1.23	0.60–2.75	0.6	1.69	0.88–3.34	0.1
ALC <600 cells per μl	7.43	3.22–16.41	<0.0001	3.95	1.83–7.83	<0.001
ALC/AMC ⩾1.1 at diagnosis	0.09	0.04–0.20	<0.0001	0.26	0.14–0.48	<0.0001
Hgb <10.5 g/dl	1.18	0.39–2.87	0.7	1.10	0.45–2.33	0.8
IPS factors ⩾3	3.30	1.54–7.11	<0.002	2.86	1.53–5.28	<0.001
Limited disease	0.48	0.20–1.05	0.07	0.34	0.16–0.67	<0.001
Male	2.26	1.04–5.28	<0.04	1.11	0.97–3.46	0.06
PET-scan negative	0.33	0.09–1.54	0.1	0.13	0.05–0.37	<0.0003
CT+RT versus CT alone	5.72	2.58–13.90	<0.0001	5.72	2.58–13.90	<0.0001
Stage 4	1.31	0.51–2.96	0.6	1.75	0.88–3.32	0.1
WBC ⩾15 cells per μl	1.88	0.56–11.68	0.3	1.84	0.66–7.60	0.3
ALC/AMC cycle 1A ⩾1.1	0.09	0.04–0.20	<0.0001	0.15	0.08–0.29	<0.0001
ALC/AMC cycle 1B ⩾1.1	0.05	0.02–0.12	<0.0001	0.10	0.5–0.20	<0.0001
ALC/AMC cycle 2A ⩾1.1	0.09	0.04–0.19	<0.0001	0.13	0.07–0.25	<0.0001
ALC/AMC cycle 2B ⩾1.1	0.10	0.04–0.21	<0.0001	0.15	0.07–0.28	<0.0001
ALC/AMC cycle 3A ⩾1.1	0.07	0.02–0.18	<0.0001	0.21	0.06–0.24	<0.0001
ALC/AMC cycle 3B ⩾1.1	0.13	0.05–0.33	<0.0001	0.15	0.07–0.29	<0.0001
ALC/AMC cycle 4A ⩾1.1	0.15	0.06–0.38	<0.0001	0.22	0.11–0.43	<0.0001
ALC/AMC cycle 4B ⩾1.1	0.12	0.04–0.30	<0.0001	0.24	0.12–0.47	<0.0001
ALC/AMC cycle 5A ⩾1.1	0.08	0.02–0.23	<0.0001	0.08	0.03–0.18	<0.0001
ALC/AMC cycle 5B ⩾1.1	0.13	0.04–0.36	<0.0001	0.19	0.08–0.40	<0.0001
ALC/AMC cycle 6A ⩾1.1	0.13	0.03–0.39	<0.0001	0.25	0.12–0.55	<0.0006
ALC/AMC cycle 6B ⩾1.1	0.09	0.02–0.28	<0.0001	0.18	0.08–0.39	<0.0001
Any cycles ⩾1.1 versus all cycles<1.1 (ALC/AMC ratio)	0.09	0.04–0.19	<0.0001	0.08	0.04–0.14	<0.0001

Abbreviations: ALC, absolute lymphocyte count; AMC, absolute monocyte count; CI, confidence interval; CT, chemotherapy; Hgb, hemoglobin; HR, hazard ratio; IPS, International Prognostic Score; OS, overall survival; PET, positron emission tomography; PFS, progression-free survival; RT, radiation therapy; WBC, white blood cell count.

**Table 4:** Multivariate analysis for OS, lymphoma-specific survival, PFS and time to progression

*Variables*	*OS*			*PFS*		
	*HR*	*95% CI*	P*-value*	*HR*	*95% CI*	P*-value*
Age >45 years	5.69	2.29–15.58	<0.0002	4.42	1.20–18.07	<0.03
ALC <600 cells per μl	3.64	1.16–11.99	<0.03	1.38	0.26–8.77	0.7
ALC/AMC ⩾1.1 at diagnosis	0.24	0.07–0.84	<0.03	0.04	0.02–0.97	<0.05
Hgb <10.5 g/dl	1.41	0.39–4.39	0.6			
IPS factors ⩾3	2.14	0.44–10.29	0.3	1.30	0.31–5.20	0.7
Limited disease				0.14	0.02–0.65	<0.01
Male	1.24	0.33–4.21	0.7			
Radiation (yes)	0.27	0.05–1.06	0.06	0.18	0.09–1.79	0.1
Any cycles ⩾1.1 versus all cycles<1.1 (ALC/AMC ratio)	0.14	0.04–0.40	<0.0002	0.19	0.05–0.82	<0.03

Abbreviations: ALC, absolute lymphocyte count; AMC, absolute monocyte count; CI, confidence interval; CT, chemotherapy; Hgb, hemoglobin; HR, hazard ratio; IPS, International Prognostic Score; OS, overall survival; PET, positron emission tomography; PFS, progression-free survival; RT, radiation therapy; WBC, white blood cell count.

## Supplementary Material

Supplementay Figure

## References

[bib1] Porrata LF Ristow K Colgan JP Habermann TM Witzig TE Inwards DJ Peripheral blood lymphocyte/monocyte ratio at diagnosis and survival in classical Hodgkin's lymphoma Hematologica 2012 97 262 269 10.3324/haematol.2011.050138 PMC3269488 21993683

[bib2] Koh YW Kang HJ Park C Yoon DH Kim S Suh C The ratio of the absolute lymphocyte count to the absolute monocyte count is associated with prognosis in Hodgkin's lymphoma: correlation with tumor-associated macrophages Oncologist 2012 17 871 880 22588324 10.1634/theoncologist.2012-0034 PMC3380887

[bib3] Cox DR Habermann TM Payne BA Klee GC Pierre RV Evaluation of the Coulter counter model S-Plus IV Am J Clin Pathol 1985 84 297 306 4036859 10.1093/ajcp/84.3.297

[bib4] Hasenclever D Diehl VA A prognostic score for advanced Hodgkin disease N Engl J Med 1998 339 1506 1514 9819449 10.1056/NEJM199811193392104

[bib5] Cheson BD Pfistner B Juweid ME Gascoyne RD Specht L Horning SJ Revised response criteria for malignant lymphoma J Clin Oncol 2007 25 579 586 17242396 10.1200/JCO.2006.09.2403

[bib6] Kaplan E Meier P Nonparametric estimation from incomplete observations J Am Stat Assoc 1958 53 457 481

[bib7] Cox DR Regression models and life-tables J R Stat Soc Ser B 1972 34 187 202

[bib8] Sun D Elson P Liedtke M Medeiros BC Earl M Alizadeh A Absolute lymphocyte count at day 28 independently predicts event-free and overall survival in adults with newly diagnosed acute lymphoblastic leukemia Am J Hematol 2012 87 957 960 22729847 10.1002/ajh.23279

[bib9] Behl D Porrata LF Markovic SN Letendre L Pruthi PK Hook CC Absolute lymphocyte count recovery after induction chemotherapy predicts superior survival in acute myelogenous leukemia Leukemia 2006 20 29 34 16281063 10.1038/sj.leu.2404032

[bib10] Chae YS Shin H Sohn SK Lee SJ Moon JH Kang BW Absolute lymphocyte count at day+21 predicts survival in patients with early-stage diffuse large B-cell lymphoma treated with rituximab, cyclophosphamide, Adriamycin, vincristine and prednisone Leuk Lymph 2012 53 1757 1763 10.3109/10428194.2012.670231 22372848

[bib11] Porrata LF Ristow K Habermann TM Ozsan N Dogan A Macon W Absolute monocyte/lymphocyte count prognostic score is independent of immunohistochemically determined cell of origin in predicting survival in diffuse large B-cell lymphoma Leuk Lymph 2012 53 2159 2165 10.3109/10428194.2012.690605 22551474

[bib12] Steidl C Lee T Shah SP Farinha P Han G Nayar T Tumor-associated macrophages and survival in classical Hodgkin's lymphoma N Engl J Med 2010 362 875 885 20220182 10.1056/NEJMoa0905680 PMC2897174

[bib13] Ribatti D Nico B Crivellato E Vacca A Macrophages and tumor angiogenesis Leukemia 2007 21 2085 2089 17878921 10.1038/sj.leu.2404900

[bib14] Green CE Liu T Montel V Hsiao G Lester RD Subramaniam S Chemoattractant signaling between tumor cells and macrophages regulates cancer cell migration, metastasis, and neovascularization PLoS ONE 2009 4 e6713 19696929 10.1371/journal.pone.0006713 PMC2725301

[bib15] Roca H Varsos ZS Sud S Craig MJ Ying C Piena KJ CCL2 and interleukin 6 promote survival of human CD11 b+ peripheral blood mononuclear cells and induce M2-type macrophages polarization J Biol Chem 2009 284 34342 34354 19833726 10.1074/jbc.M109.042671 PMC2797202

[bib16] Dirkx AE Oude Egbrink MG Wagstaff J Griffioen AW Monocyte/macrophages infiltration in tumors modulators of angiogenesis J Leukocyte Bio 2006 80 1183 1196 16997855 10.1189/jlb.0905495

