# Supplementary material for: Peripheral blood absolute lymphocyte/monocyte ratio recovery during ABVD treatment cycles predicts clinical outcomes in classical Hodgkin lymphoma
Source: Blood Cancer J. 2013 Apr 19;3(4):e110–. doi: 10.1038/bcj.2013.8 (PMC3641323; doi:10.1038/bcj.2013.8)
Supplement: Supplementay Figure [file bcj20138x1.ppt]

## Slide 1
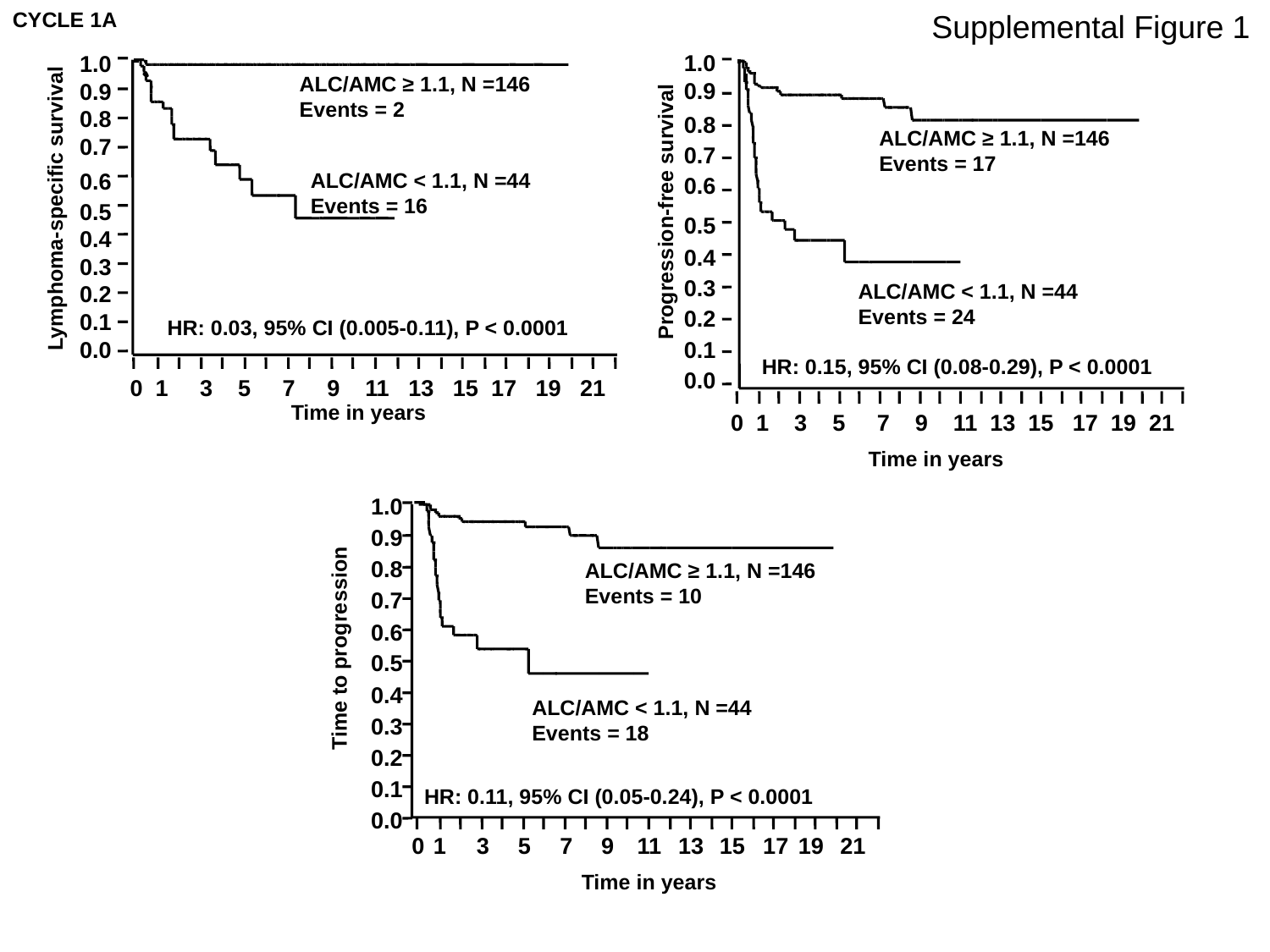

CYCLE 1A
Supplemental Figure 1
1.0
0.9
0.8
ALC/AMC ≥ 1.1, N =146
Events = 17
0.7
0.6
Progression-free survival
0.5
0.4
0.3
ALC/AMC < 1.1, N =44
Events = 24
0.2
0.1
HR: 0.15, 95% CI (0.08-0.29), P < 0.0001
0.0
0 1 3 5 7 9 11 13 15 17 19 21
Time in years
1.0
ALC/AMC ≥ 1.1, N =146
Events = 2
0.9
0.8
0.7
0.6
ALC/AMC < 1.1, N =44
Events = 16
Lymphoma-specific survival
0.5
0.4
0.3
0.2
0.1
HR: 0.03, 95% CI (0.005-0.11), P < 0.0001
0.0
 0 1 3 5 7 9 11 13 15 17 19 21
Time in years
1.0
0.9
ALC/AMC ≥ 1.1, N =146
Events = 10
0.8
0.7
0.6
Time to progression
0.5
0.4
ALC/AMC < 1.1, N =44
Events = 18
0.3
0.2
0.1
HR: 0.11, 95% CI (0.05-0.24), P < 0.0001
0.0
0
 1
 3
 5
 7
 9
 11
 13
 15
 17
19
21
Time in years

## Slide 2
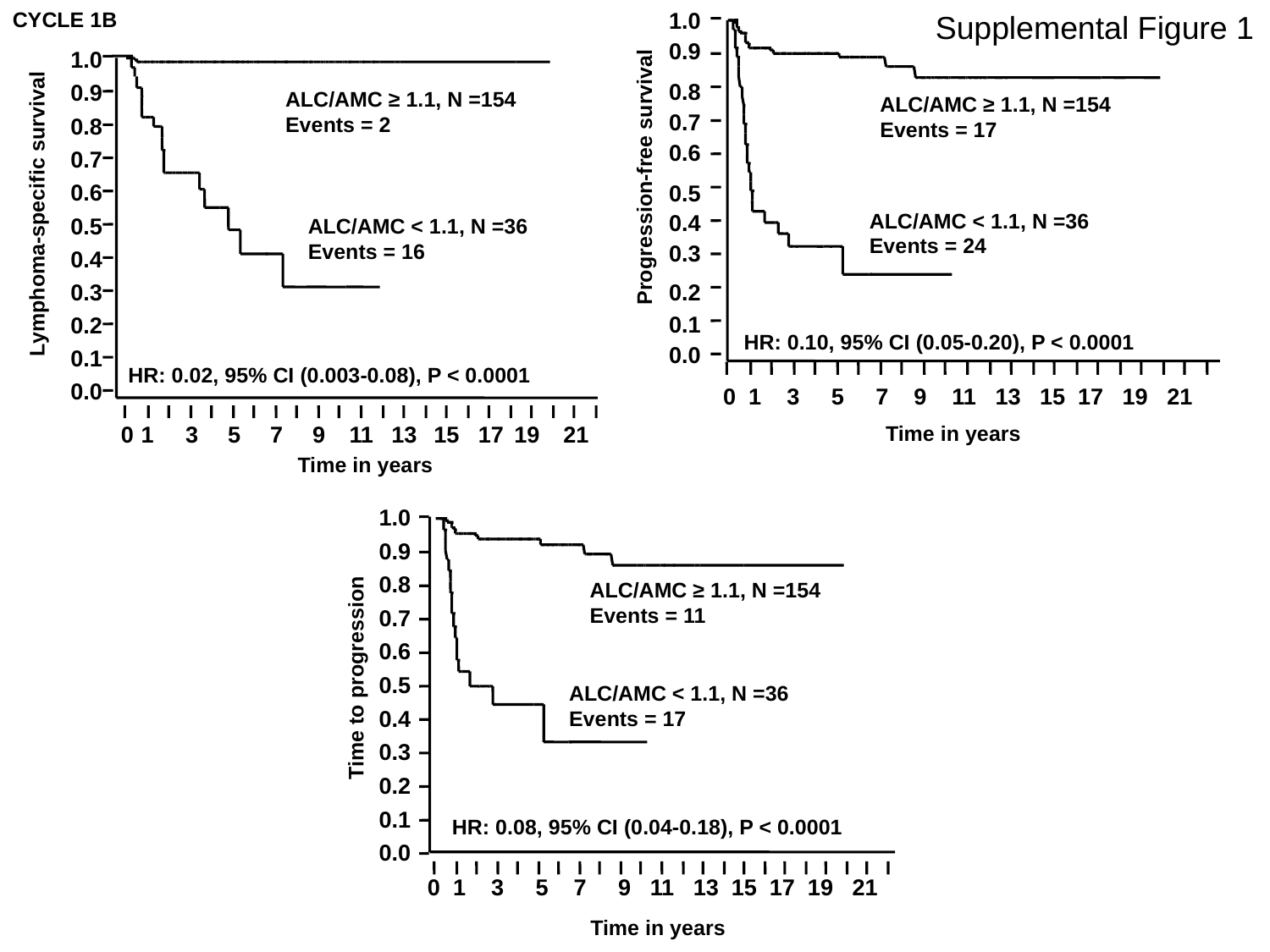

CYCLE 1B
1.0
0.9
0.8
ALC/AMC ≥ 1.1, N =154
Events = 17
0.7
0.6
Progression-free survival
0.5
ALC/AMC < 1.1, N =36
Events = 24
0.4
0.3
0.2
0.1
HR: 0.10, 95% CI (0.05-0.20), P < 0.0001
0.0
0 1 3 5 7 9 11 13 15 17 19 21
Time in years
Supplemental Figure 1
1.0
0.9
ALC/AMC ≥ 1.1, N =154
Events = 2
0.8
0.7
0.6
Lymphoma-specific survival
ALC/AMC < 1.1, N =36
Events = 16
0.5
0.4
0.3
0.2
0.1
HR: 0.02, 95% CI (0.003-0.08), P < 0.0001
0.0
0
 1
 3
 5
 7
 9
 11
 13
 15
 17
19
 21
Time in years
1.0
0.9
0.8
ALC/AMC ≥ 1.1, N =154
Events = 11
0.7
0.6
Time to progression
0.5
ALC/AMC < 1.1, N =36
Events = 17
0.4
0.3
0.2
0.1
HR: 0.08, 95% CI (0.04-0.18), P < 0.0001
0.0
 0 1 3 5 7 9 11 13 15 17 19 21
Time in years

## Slide 3
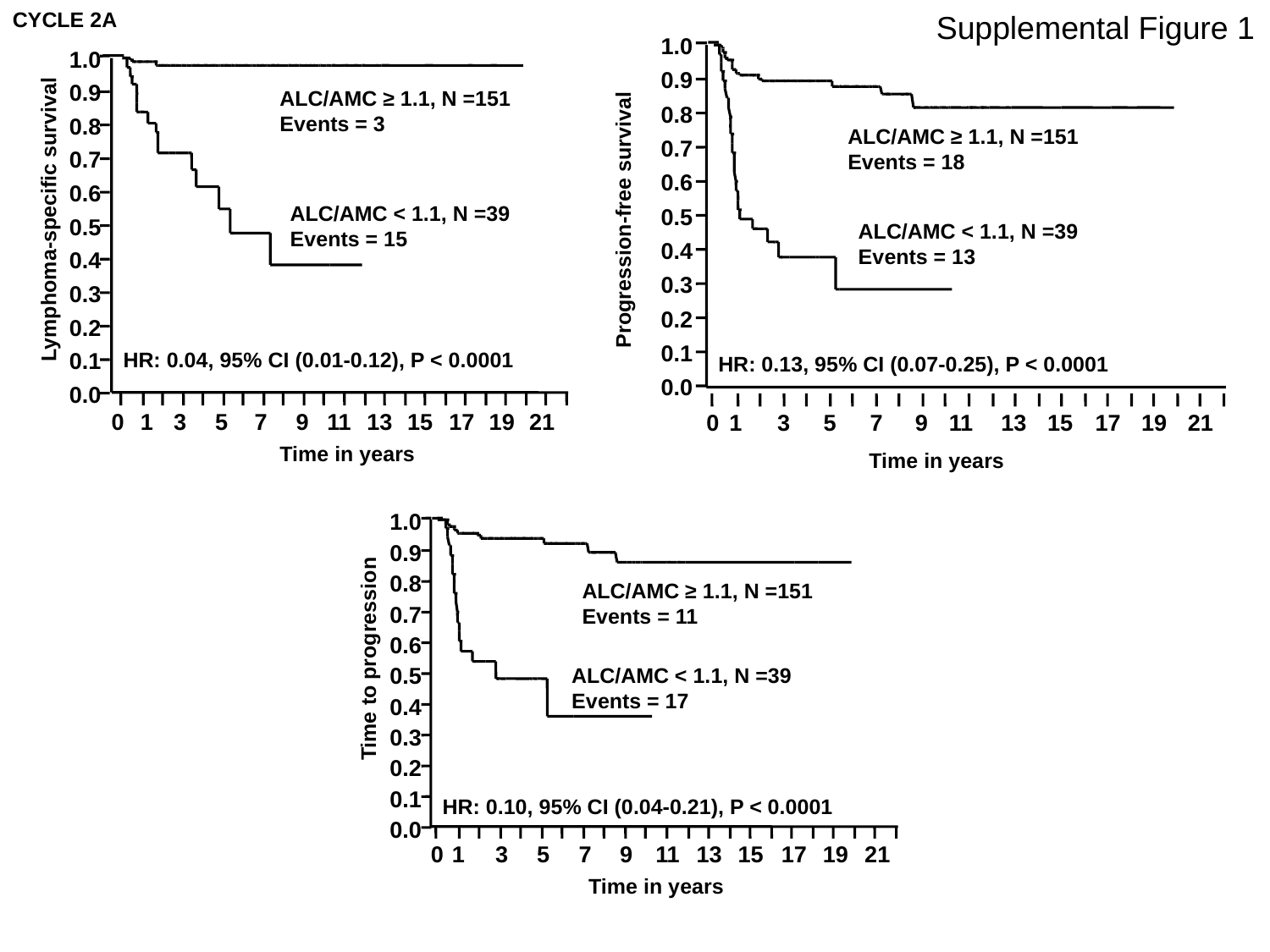

CYCLE 2A
Supplemental Figure 1
1.0
0.9
0.8
ALC/AMC ≥ 1.1, N =151
Events = 18
0.7
0.6
Progression-free survival
0.5
ALC/AMC < 1.1, N =39
Events = 13
0.4
0.3
0.2
0.1
HR: 0.13, 95% CI (0.07-0.25), P < 0.0001
0.0
0
 1
 3
 5
 7
 9
11
 13
 15
 17
 19
 21
Time in years
1.0
0.9
ALC/AMC ≥ 1.1, N =151
Events = 3
0.8
0.7
0.6
ALC/AMC < 1.1, N =39
Events = 15
Lymphoma-specific survival
0.5
0.4
0.3
0.2
HR: 0.04, 95% CI (0.01-0.12), P < 0.0001
0.1
0.0
0
1
3
5
7
9
11
13
15
17
19
21
Time in years
1.0
0.9
0.8
ALC/AMC ≥ 1.1, N =151
Events = 11
0.7
0.6
Time to progression
ALC/AMC < 1.1, N =39
Events = 17
0.5
0.4
0.3
0.2
0.1
HR: 0.10, 95% CI (0.04-0.21), P < 0.0001
0.0
0
 1
 3
 5
 7
 9
 11
 13
 15
 17
 19
 21
Time in years

## Slide 4
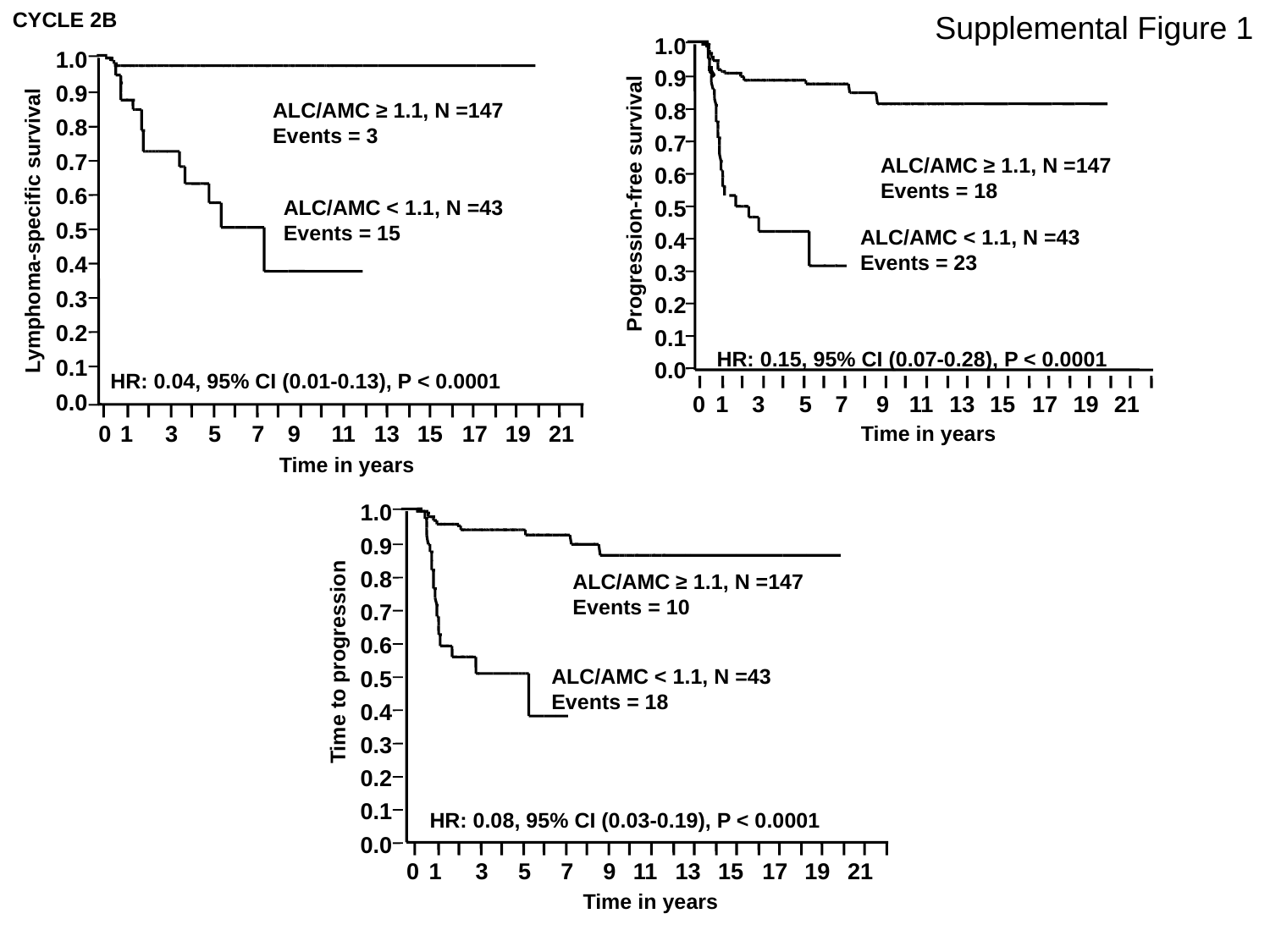

CYCLE 2B
Supplemental Figure 1
1.0
1.0
0.9
ALC/AMC ≥ 1.1, N =147
Events = 3
0.8
0.7
0.6
ALC/AMC < 1.1, N =43
Events = 15
Lymphoma-specific survival
0.5
0.4
0.3
0.2
0.1
HR: 0.04, 95% CI (0.01-0.13), P < 0.0001
0.0
0
 1
 3
 5
 7
 9
 11
 13
 15
 17
 19
 21
Time in years
0.9
0.8
0.7
ALC/AMC ≥ 1.1, N =147
Events = 18
0.6
Progression-free survival
0.5
ALC/AMC < 1.1, N =43
Events = 23
0.4
0.3
0.2
0.1
HR: 0.15, 95% CI (0.07-0.28), P < 0.0001
0.0
0
 1
3
 5
7
 9
11
13
15
17
19
21
Time in years
1.0
0.9
ALC/AMC ≥ 1.1, N =147
Events = 10
0.8
0.7
0.6
Time to progression
ALC/AMC < 1.1, N =43
Events = 18
0.5
0.4
0.3
0.2
0.1
HR: 0.08, 95% CI (0.03-0.19), P < 0.0001
0.0
0
1
 3
 5
 7
 9
11
13
15
17
19
21
Time in years

## Slide 5
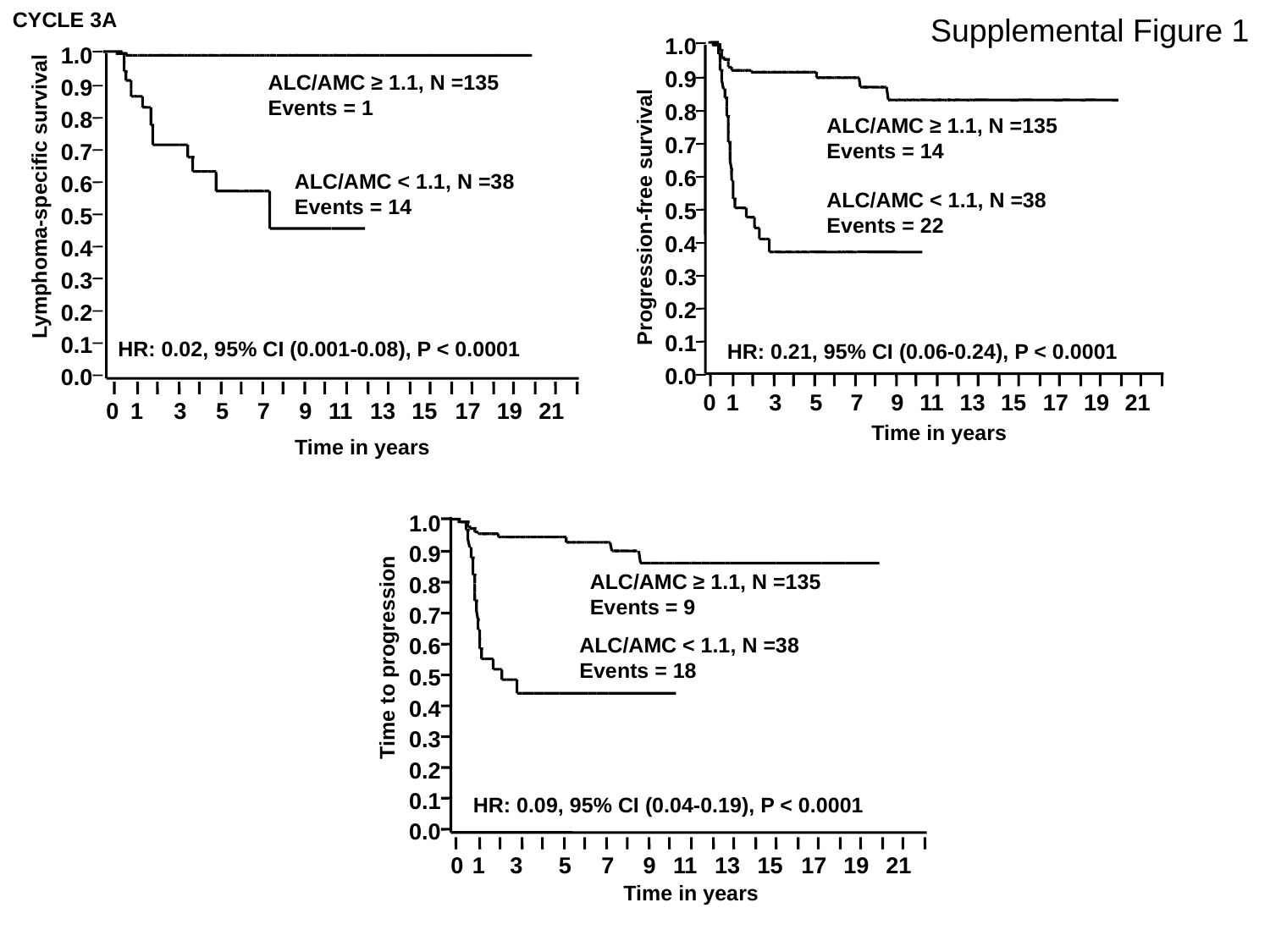

CYCLE 3A
Supplemental Figure 1
1.0
0.9
0.8
ALC/AMC ≥ 1.1, N =135
Events = 14
0.7
0.6
ALC/AMC < 1.1, N =38
Events = 22
0.5
Progression-free survival
0.4
0.3
0.2
0.1
HR: 0.21, 95% CI (0.06-0.24), P < 0.0001
0.0
0
 1
 3
 5
 7
 9
11
13
15
17
19
21
Time in years
1.0
ALC/AMC ≥ 1.1, N =135
Events = 1
0.9
0.8
0.7
ALC/AMC < 1.1, N =38
Events = 14
0.6
Lymphoma-specific survival
0.5
0.4
0.3
0.2
HR: 0.02, 95% CI (0.001-0.08), P < 0.0001
0.1
0.0
0
 1
 3
 5
 7
 9
11
13
15
17
19
21
Time in years
1.0
0.9
ALC/AMC ≥ 1.1, N =135
Events = 9
0.8
0.7
ALC/AMC < 1.1, N =38
Events = 18
0.6
Time to progression
0.5
0.4
0.3
0.2
HR: 0.09, 95% CI (0.04-0.19), P < 0.0001
0.1
0.0
0
 1
3
 5
 7
 9
11
13
15
17
19
21
Time in years

## Slide 6
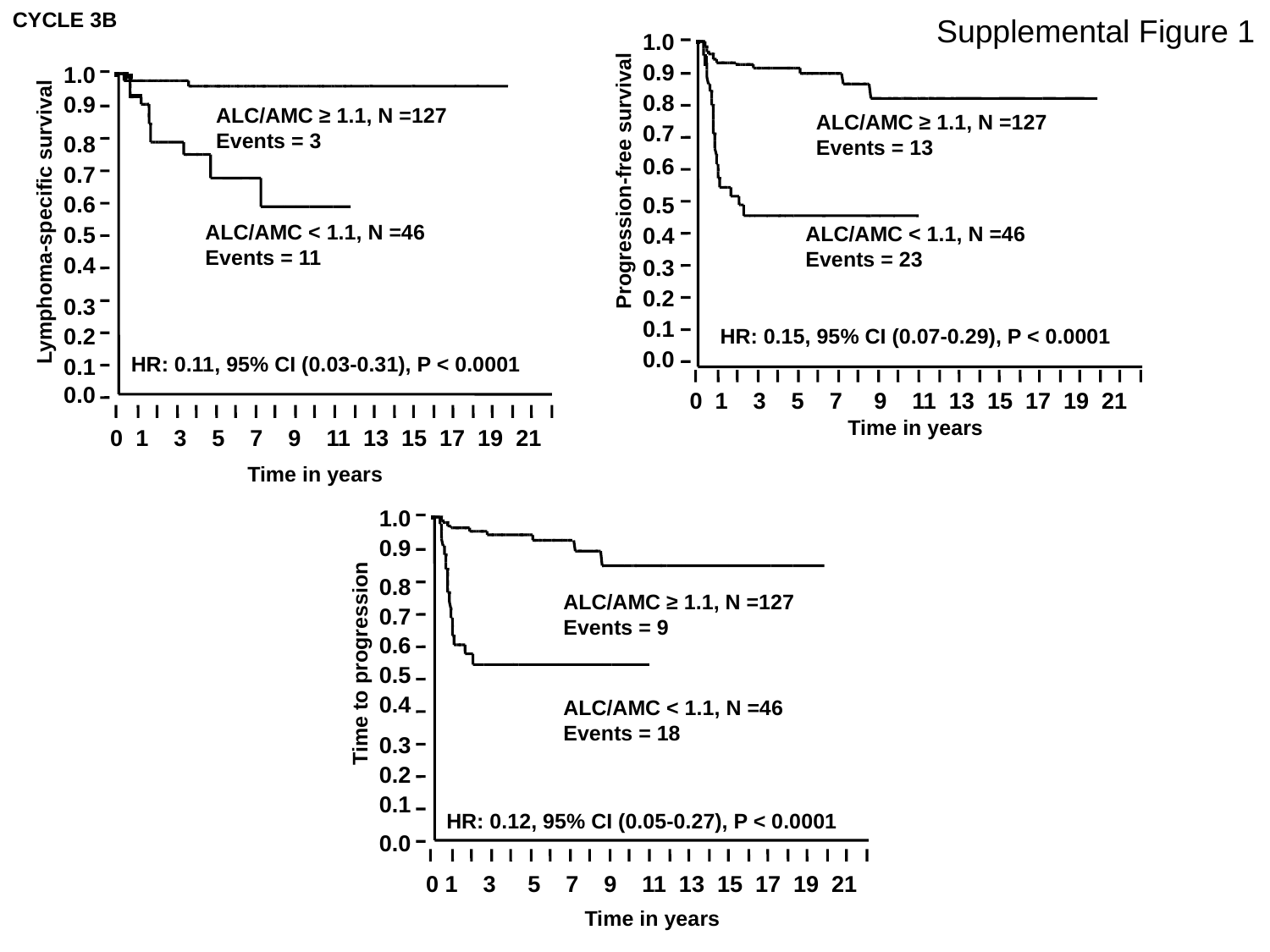

CYCLE 3B
Supplemental Figure 1
1.0
0.9
0.8
ALC/AMC ≥ 1.1, N =127
Events = 13
0.7
0.6
Progression-free survival
0.5
ALC/AMC < 1.1, N =46
Events = 23
0.4
0.3
0.2
0.1
HR: 0.15, 95% CI (0.07-0.29), P < 0.0001
0.0
0 1 3 5 7 9 11 13 15 17 19 21
Time in years
1.0
0.9
ALC/AMC ≥ 1.1, N =127
Events = 3
0.8
0.7
0.6
Lymphoma-specific survival
ALC/AMC < 1.1, N =46
Events = 11
0.5
0.4
0.3
0.2
HR: 0.11, 95% CI (0.03-0.31), P < 0.0001
0.1
0.0
0 1 3 5 7 9 11 13 15 17 19 21
Time in years
1.0
0.9
0.8
ALC/AMC ≥ 1.1, N =127
Events = 9
0.7
0.6
Time to progression
0.5
0.4
ALC/AMC < 1.1, N =46
Events = 18
0.3
0.2
0.1
HR: 0.12, 95% CI (0.05-0.27), P < 0.0001
0.0
0 1 3 5 7 9 11 13 15 17 19 21
Time in years

## Slide 7
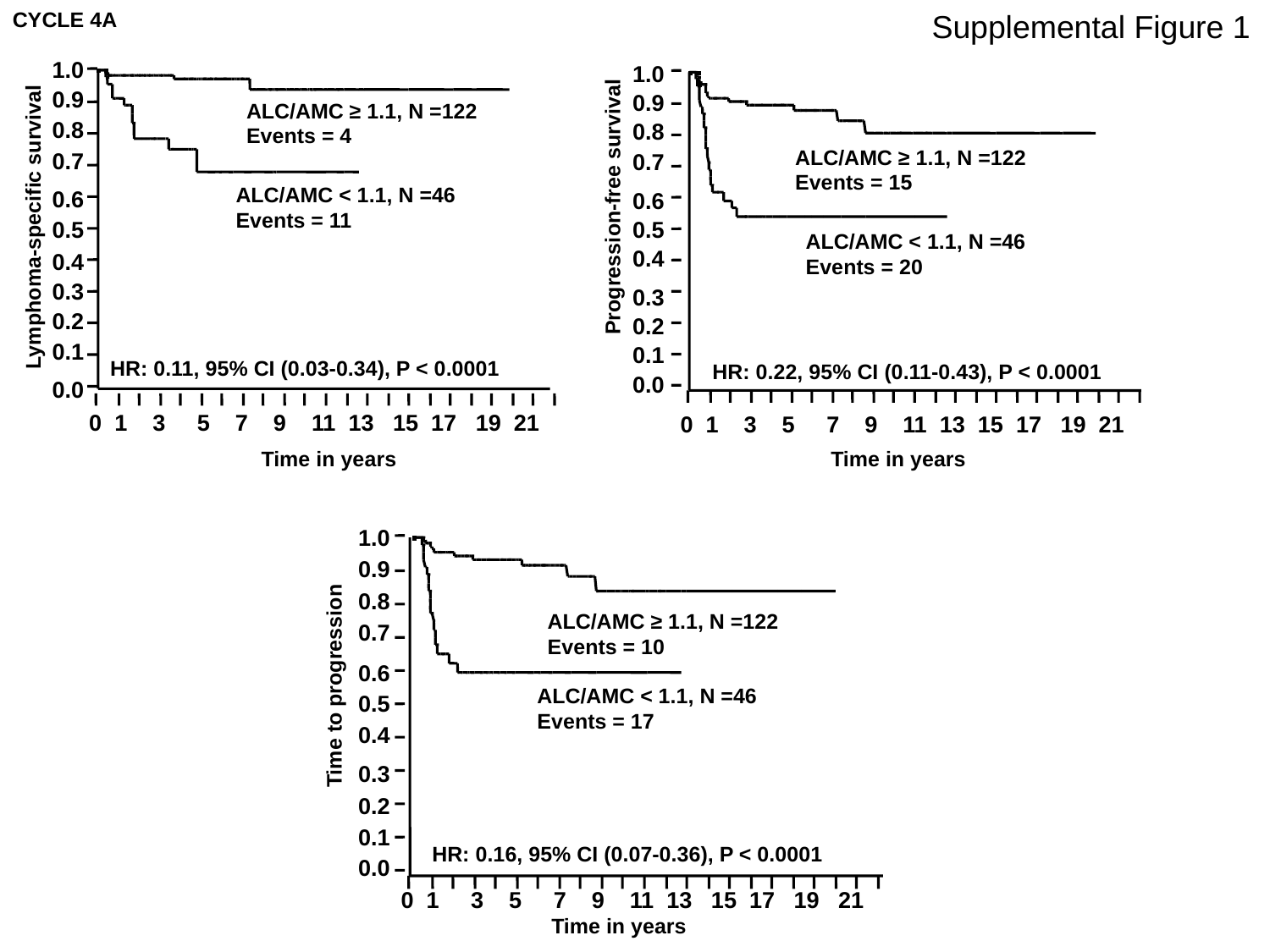

CYCLE 4A
Supplemental Figure 1
1.0
0.9
ALC/AMC ≥ 1.1, N =122
Events = 4
0.8
0.7
ALC/AMC < 1.1, N =46
Events = 11
0.6
Lymphoma-specific survival
0.5
0.4
0.3
0.2
0.1
HR: 0.11, 95% CI (0.03-0.34), P < 0.0001
0.0
0 1 3 5 7 9 11 13 15 17 19 21
Time in years
1.0
0.9
0.8
ALC/AMC ≥ 1.1, N =122
Events = 15
0.7
0.6
Progression-free survival
0.5
ALC/AMC < 1.1, N =46
Events = 20
0.4
0.3
0.2
0.1
HR: 0.22, 95% CI (0.11-0.43), P < 0.0001
0.0
0 1 3 5 7 9 11 13 15 17 19 21
Time in years
1.0
0.9
0.8
0.7
0.6
Time to progression
0.5
0.4
0.3
0.2
0.1
HR: 0.16, 95% CI (0.07-0.36), P < 0.0001
0.0
0 1 3 5 7 9 11 13 15 17 19 21
Time in years
ALC/AMC ≥ 1.1, N =122
Events = 10
ALC/AMC < 1.1, N =46
Events = 17

## Slide 8
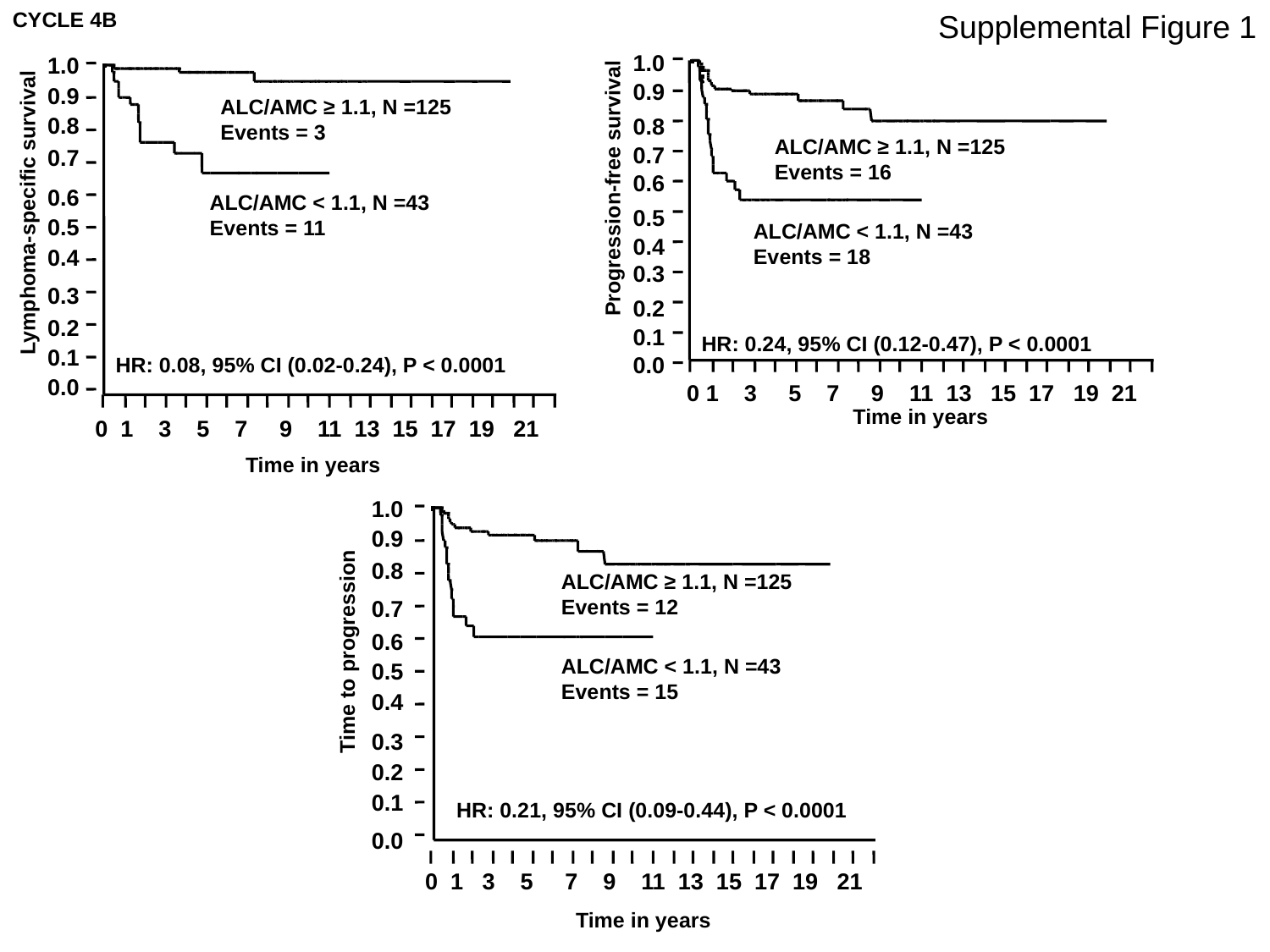

CYCLE 4B
Supplemental Figure 1
1.0
0.9
0.8
ALC/AMC ≥ 1.1, N =125
Events = 16
0.7
0.6
Progression-free survival
0.5
ALC/AMC < 1.1, N =43
Events = 18
0.4
0.3
0.2
0.1
HR: 0.24, 95% CI (0.12-0.47), P < 0.0001
0.0
 0 1 3 5 7 9 11 13 15 17 19 21
Time in years
1.0
0.9
ALC/AMC ≥ 1.1, N =125
Events = 3
0.8
0.7
0.6
ALC/AMC < 1.1, N =43
Events = 11
Lymphoma-specific survival
0.5
0.4
0.3
0.2
0.1
HR: 0.08, 95% CI (0.02-0.24), P < 0.0001
0.0
0 1 3 5 7 9 11 13 15 17 19 21
Time in years
1.0
0.9
0.8
ALC/AMC ≥ 1.1, N =125
Events = 12
0.7
0.6
Time to progression
ALC/AMC < 1.1, N =43
Events = 15
0.5
0.4
0.3
0.2
0.1
HR: 0.21, 95% CI (0.09-0.44), P < 0.0001
0.0
0 1 3 5 7 9 11 13 15 17 19 21
Time in years

## Slide 9
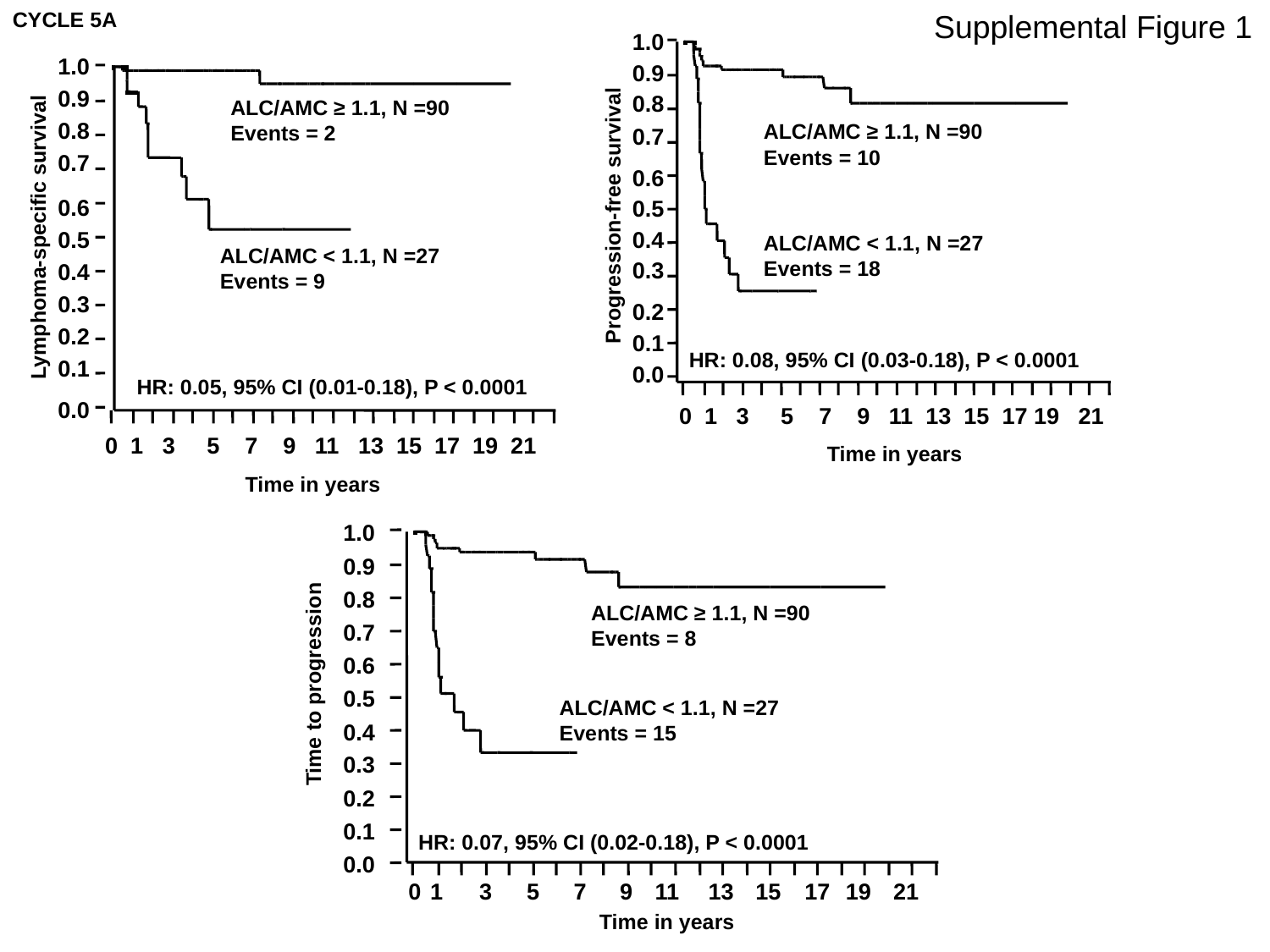

CYCLE 5A
Supplemental Figure 1
1.0
0.9
0.8
ALC/AMC ≥ 1.1, N =90
Events = 10
0.7
0.6
0.5
Progression-free survival
0.4
ALC/AMC < 1.1, N =27
Events = 18
0.3
0.2
0.1
HR: 0.08, 95% CI (0.03-0.18), P < 0.0001
0.0
0 1 3 5 7 9 11 13 15 17 19 21
Time in years
1.0
0.9
ALC/AMC ≥ 1.1, N =90
Events = 2
0.8
0.7
0.6
Lymphoma-specific survival
0.5
ALC/AMC < 1.1, N =27
Events = 9
0.4
0.3
0.2
0.1
HR: 0.05, 95% CI (0.01-0.18), P < 0.0001
0.0
0 1 3 5 7 9 11 13 15 17 19 21
Time in years
1.0
0.9
0.8
ALC/AMC ≥ 1.1, N =90
Events = 8
0.7
0.6
Time to progression
0.5
ALC/AMC < 1.1, N =27
Events = 15
0.4
0.3
0.2
0.1
HR: 0.07, 95% CI (0.02-0.18), P < 0.0001
0.0
0
 1
 3
 5
 7
 9
11
 13
 15
 17
19
21
Time in years

## Slide 10
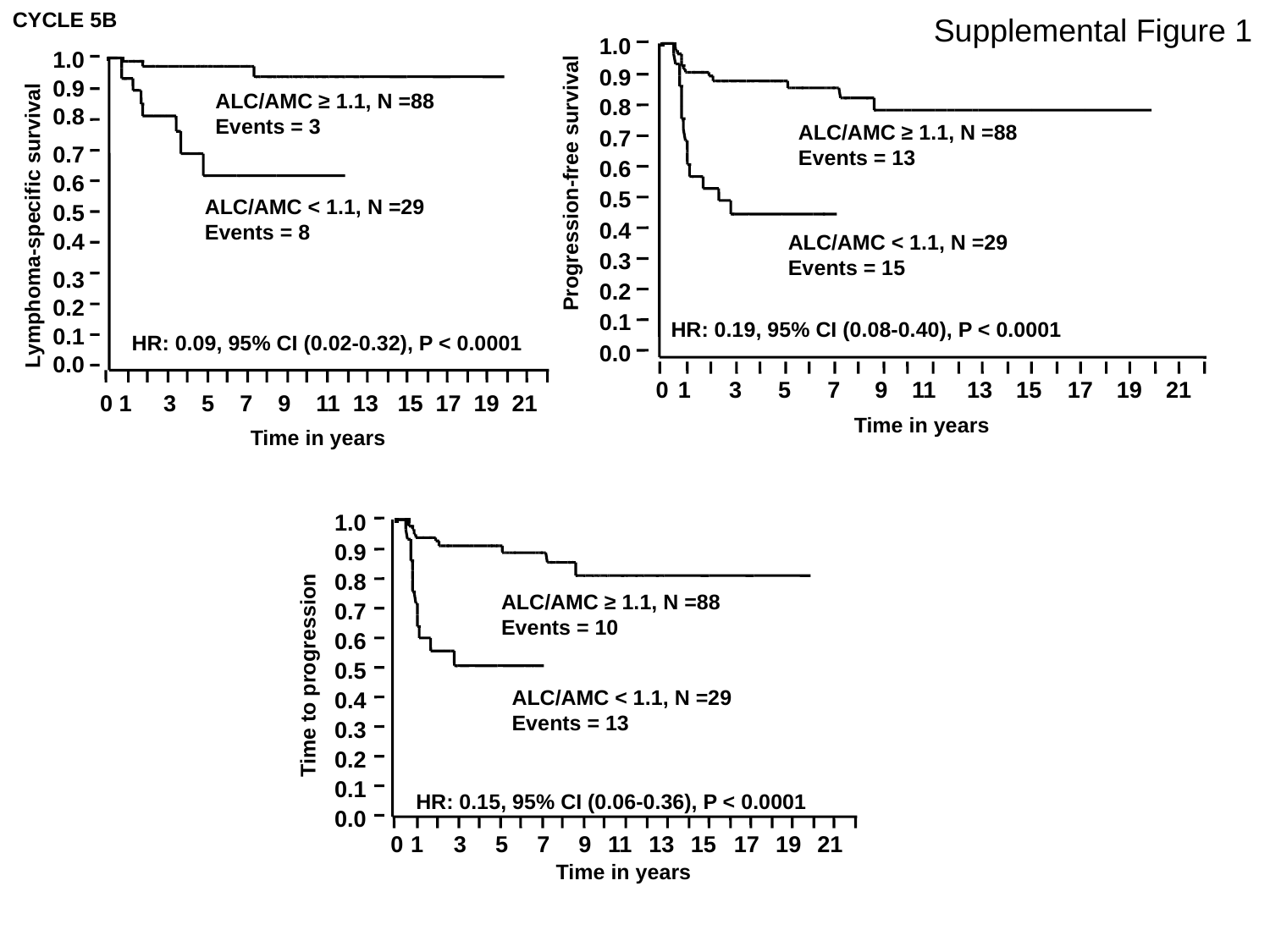

CYCLE 5B
Supplemental Figure 1
1.0
0.9
0.8
ALC/AMC ≥ 1.1, N =88
Events = 13
0.7
0.6
Progression-free survival
0.5
0.4
ALC/AMC < 1.1, N =29
Events = 15
0.3
0.2
0.1
HR: 0.19, 95% CI (0.08-0.40), P < 0.0001
0.0
0
 1
 3
 5
 7
 9
11
 13
 15
 17
 19
 21
Time in years
1.0
0.9
ALC/AMC ≥ 1.1, N =88
Events = 3
0.8
0.7
0.6
ALC/AMC < 1.1, N =29
Events = 8
0.5
Lymphoma-specific survival
0.4
0.3
0.2
0.1
HR: 0.09, 95% CI (0.02-0.32), P < 0.0001
0.0
0 1 3 5 7 9 11 13 15 17 19 21
Time in years
1.0
0.9
0.8
ALC/AMC ≥ 1.1, N =88
Events = 10
0.7
0.6
0.5
Time to progression
ALC/AMC < 1.1, N =29
Events = 13
0.4
0.3
0.2
0.1
HR: 0.15, 95% CI (0.06-0.36), P < 0.0001
0.0
0
 1
 3
 5
 7
 9
11
13
15
17
19
21
Time in years

## Slide 11
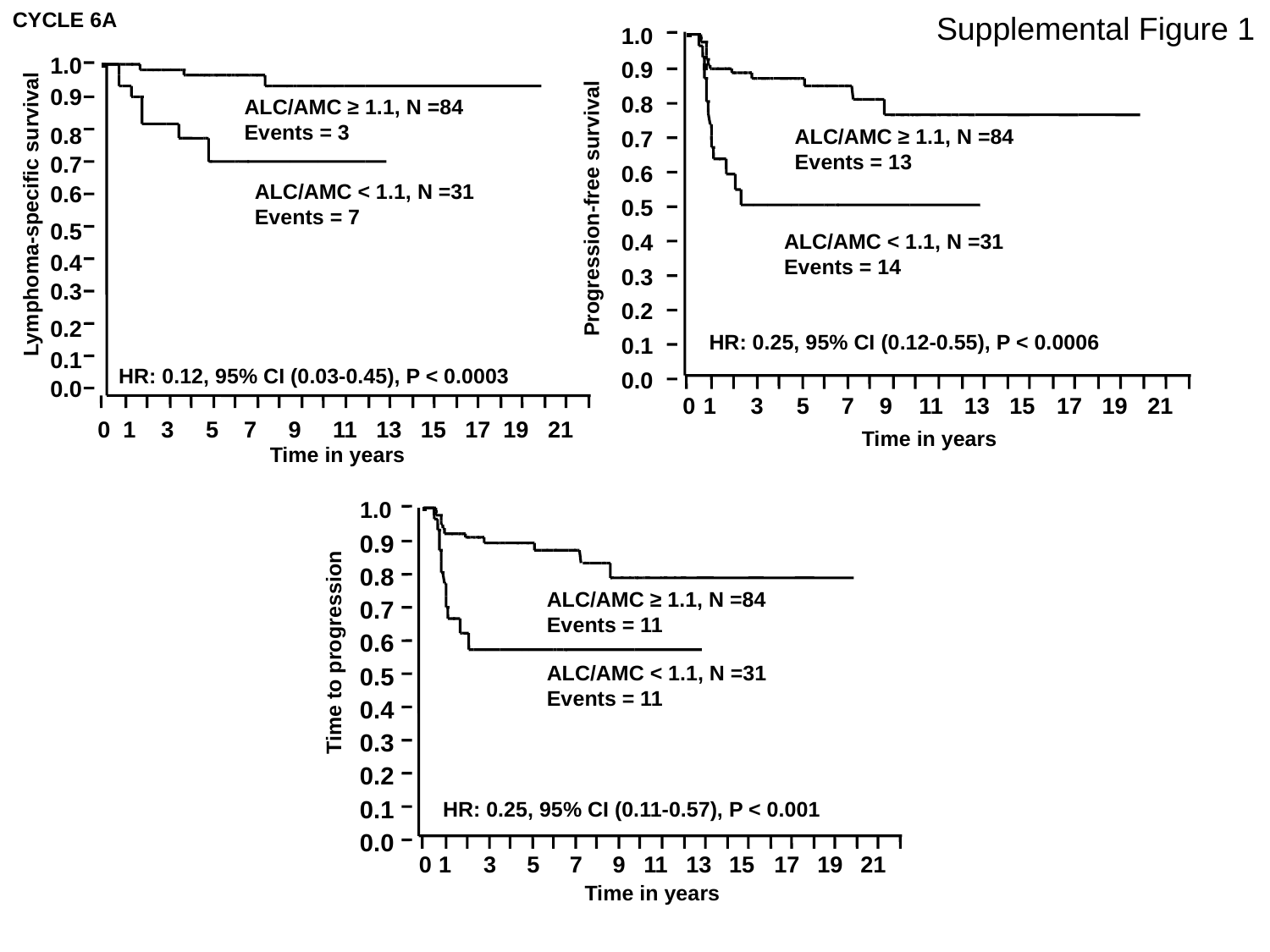

CYCLE 6A
Supplemental Figure 1
1.0
0.9
0.8
ALC/AMC ≥ 1.1, N =84
Events = 13
0.7
0.6
Progression-free survival
0.5
ALC/AMC < 1.1, N =31
Events = 14
0.4
0.3
0.2
HR: 0.25, 95% CI (0.12-0.55), P < 0.0006
0.1
0.0
0
 1
 3
 5
 7
 9
11
13
15
17
19
21
Time in years
1.0
0.9
ALC/AMC ≥ 1.1, N =84
Events = 3
0.8
0.7
ALC/AMC < 1.1, N =31
Events = 7
0.6
Lymphoma-specific survival
0.5
0.4
0.3
0.2
0.1
HR: 0.12, 95% CI (0.03-0.45), P < 0.0003
0.0
0 1 3 5 7 9 11 13 15 17 19 21
Time in years
1.0
0.9
0.8
ALC/AMC ≥ 1.1, N =84
Events = 11
0.7
0.6
Time to progression
ALC/AMC < 1.1, N =31
Events = 11
0.5
0.4
0.3
0.2
HR: 0.25, 95% CI (0.11-0.57), P < 0.001
0.1
0.0
0
 1
 3
 5
 7
 9
11
13
15
17
19
21
Time in years

## Slide 12
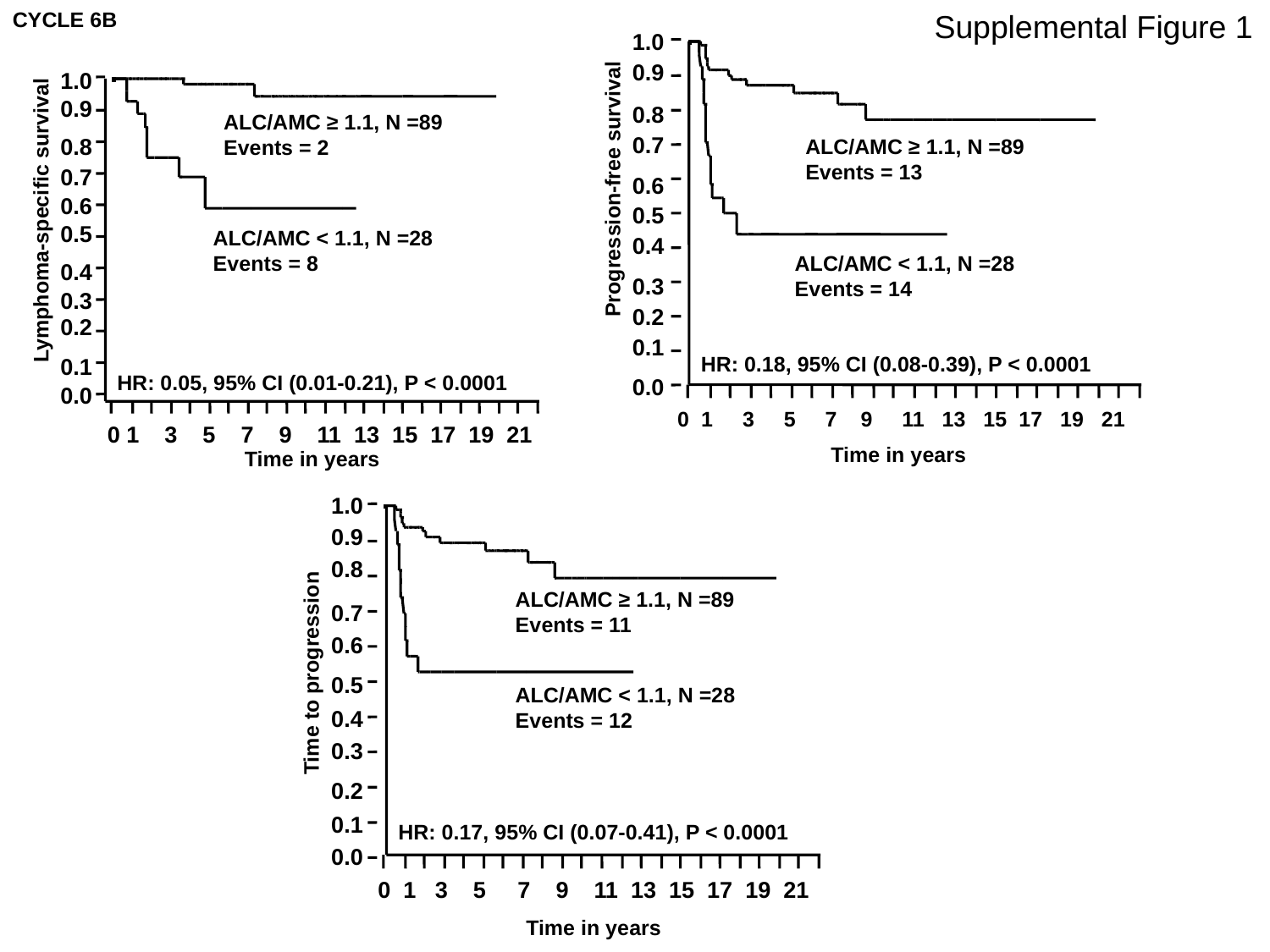

CYCLE 6B
Supplemental Figure 1
1.0
0.9
0.8
0.7
ALC/AMC ≥ 1.1, N =89
Events = 13
0.6
Progression-free survival
0.5
0.4
ALC/AMC < 1.1, N =28
Events = 14
0.3
0.2
0.1
HR: 0.18, 95% CI (0.08-0.39), P < 0.0001
0.0
0 1 3 5 7 9 11 13 15 17 19 21
Time in years
1.0
0.9
ALC/AMC ≥ 1.1, N =89
Events = 2
0.8
0.7
0.6
Lymphoma-specific survival
0.5
ALC/AMC < 1.1, N =28
Events = 8
0.4
0.3
0.2
0.1
HR: 0.05, 95% CI (0.01-0.21), P < 0.0001
0.0
0 1 3 5 7 9 11 13 15 17 19 21
Time in years
1.0
0.9
0.8
ALC/AMC ≥ 1.1, N =89
Events = 11
0.7
0.6
Time to progression
0.5
ALC/AMC < 1.1, N =28
Events = 12
0.4
0.3
0.2
0.1
HR: 0.17, 95% CI (0.07-0.41), P < 0.0001
0.0
0 1 3 5 7 9 11 13 15 17 19 21
Time in years

## Slide 13
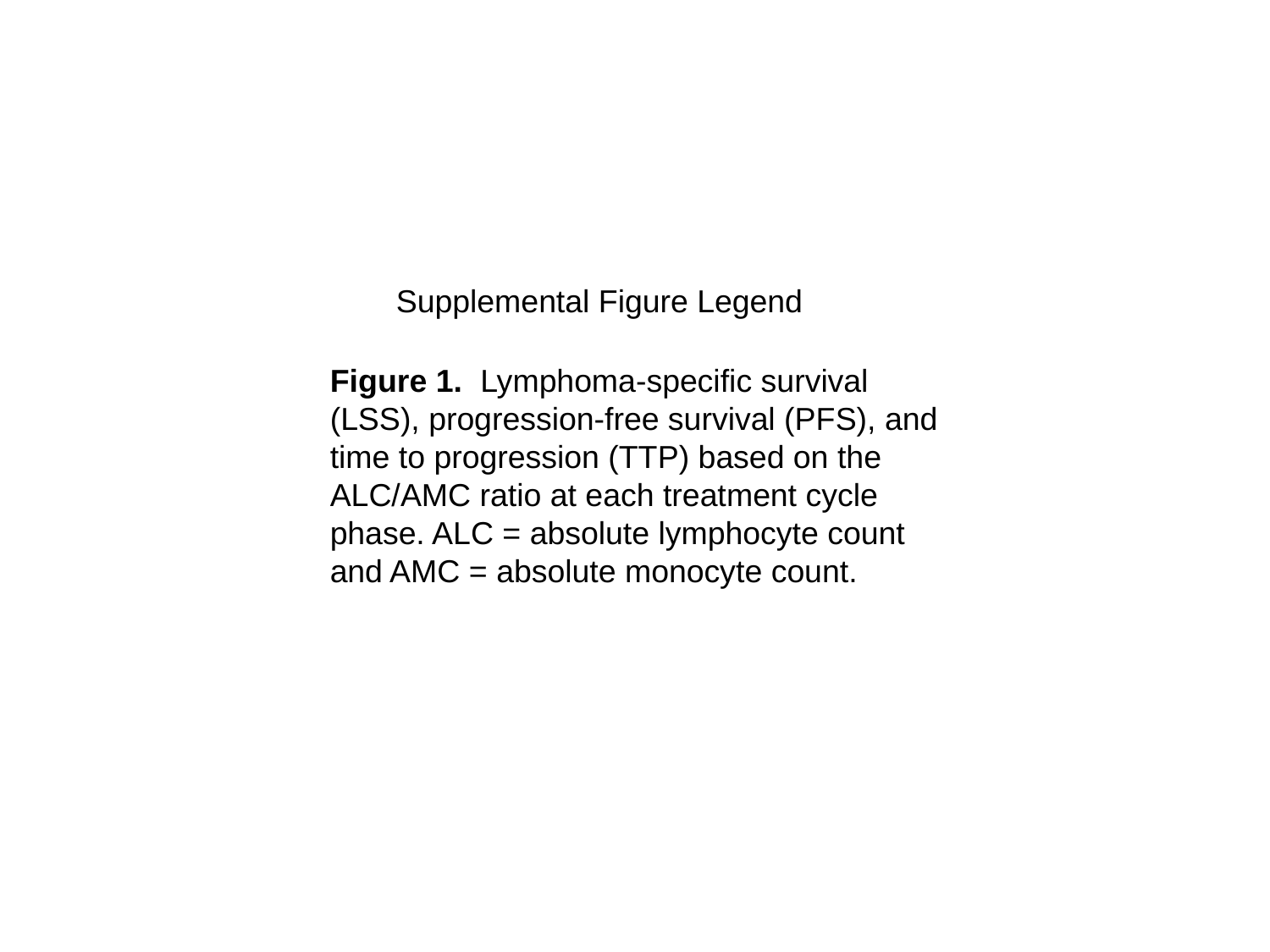

Supplemental Figure Legend
Figure 1. Lymphoma-specific survival (LSS), progression-free survival (PFS), and time to progression (TTP) based on the ALC/AMC ratio at each treatment cycle phase. ALC = absolute lymphocyte count and AMC = absolute monocyte count.
