# Supplementary material for: Peripheral blood absolute lymphocyte/monocyte ratio recovery during ABVD treatment cycles predicts clinical outcomes in classical Hodgkin lymphoma
Source: Blood Cancer J. 2013 Apr 19;3(4):e110–. doi: 10.1038/bcj.2013.8 (PMC3641323; doi:10.1038/bcj.2013.8)
Supplement: Supplementary Information [file bcj20138x2.doc]

Manuscript: MARIA13-BCJ-0002T

Supplemental File

***ALC/AMC ratio recovery at each treatment cycle and overall survival***

Patients with an ALC/AMC ratio ≥ 1.1 experienced superior OS compared with patients with an ALC/AMC ratio < 1.1 at each treatment cycle phase of ABVD (figure 1) [cycle 1A, the median was not reached versus 5.3 years, the 5 year OS rates were 96 %(95%CI, 90%-99%) versus 47% (95%CI, 30%-66%), (p <0.0001), respectively; cycle 1B, the median was not reached versus 3.5 years, the 5 year OS rates were 97 %(95%CI, 93%-100%) versus 34% (95%CI, 25%-59%), (p <0.0001), respectively; cycle 2A, the median was not reached versus 4.8 years, the 5 year OS rates were 95 %(95%CI, 90%-99%) versus 42% (95%CI, 30%-66%), (p <0.0001), respectively; cycle 2B, the median was not reached versus 5.3 years, the 5 year OS rates were 96 %(95%CI, 90%-99%) versus 51% (95%CI, 33%-66%), (p <0.0001), respectively; cycle 3A, the median was not reached versus 7.4 years, the 5 year OS rates were 97 %(95%CI, 92%-100%) versus 51% (95%CI, 40%-75%), (p <0.0001), respectively; cycle 3B, the median was not reached versus 3.4 years, the 5 year OS rates were 95 %(95%CI, 89%-99%) versus 60% (95%CI, 50%-80%), (p <0.0001), respectively; cycle 4A, the median was not reached versus 4.8 years, the 5 year OS rates were 96 %(95%CI, 90%-99%) versus 63% (95%CI, 53%-84%), (p <0.0001), respectively; cycle 4B, the median was not reached versus not reached, the 5 year OS rates were 96 %(95%CI, 92%-100%) versus 61% (95%CI, 42%-78%), (p <0.0001), respectively; cycle 5A, the median was not reached versus 4.8 years, the 5 year OS rates were 98 %(95%CI, 91%-100%) versus 47% (95%CI, 33%-74%), (p <0.0001), respectively; cycle 5B, the median was not reached versus not reached, the 5 year OS rates were 96 %(95%CI, 90%-99%) versus 56% (95%CI, 40%-80%), (p <0.0001), respectively; cycle 6A, the median was not reached versus not reached, the 5 year OS rates were 97 %(95%CI, 90%-100%) versus 64% (95%CI, 50%-85%), (p <0.0001), respectively; and cycle 6B, the median was not reached versus not reached, the 5 year OS rates were 97 %(95%CI, 90%-100%) versus 55% (95%CI, 42%-80%), (p <0.0001), respectively].
